# Recent Advances in the Application of Nitro(het)aromatic Compounds for Treating and/or Fluorescent Imaging of Tumor Hypoxia

**DOI:** 10.3390/molecules29153475

**Published:** 2024-07-25

**Authors:** Kameliya Anichina, Nikolay Lumov, Ventsislav Bakov, Denitsa Yancheva, Nikolai Georgiev

**Affiliations:** 1 Department of Organic Synthesis, University of Chemical Technology and Metallurgy, 8 Kliment Ohridski Blvd., 1756 Sofia, Bulgaria; kameliya_anichina@uctm.edu (K.A.); nikolay.lumov@orgchm.bas.bg (N.L.); bakov@uctm.edu (V.B.); denitsa.pantaleeva@orgchm.bas.bg (D.Y.); 2 Institute of Organic Chemistry with Centre of Phytochemistry, Bulgarian Academy of Sciences, Acad. G. Bonchev str. Bl. 9, 1113 Sofia, Bulgaria

**Keywords:** nitroaromatic compounds, hypoxia, tumor treatment, fluorescence imaging

## Abstract

This review delves into recent advancements in the field of nitro(het)aromatic bioreductive agents tailored for hypoxic environments. These compounds are designed to exploit the low-oxygen conditions typically found in solid tumors, making them promising candidates for targeted cancer therapies. Initially, this review focused on their role as gene-directed enzyme prodrugs, which are inert until activated by specific enzymes within tumor cells. Upon activation, these prodrugs undergo chemical transformations that convert them into potent cytotoxic agents, selectively targeting cancerous tissue while sparing healthy cells. Additionally, this review discusses recent developments in prodrug conjugates containing nitro(het)aromatic moieties, designed to activate under low-oxygen conditions within tumors. This approach enhances their efficacy and specificity in cancer treatment. Furthermore, this review covers innovative research on using nitro(het)aromatic compounds as fluorescent probes for imaging hypoxic tumors. These probes enable non-invasive visualization of low-oxygen regions within tumors, providing valuable insights for the diagnosis, treatment planning, and monitoring of therapeutic responses. We hope this review will inspire researchers to design and synthesize improved compounds for selective cancer treatment and early diagnostics.

## 1. Introduction

Hypoxia, a condition of low oxygen levels ranging from 0.02 to 2%, is a physiological characteristic of most solid tumors. It occurs because the tumor’s rapid growth outstrips the oxygen supply and is compounded by impaired blood flow due to the formation of abnormal blood vessels supplying the tumor [1]. 

Severe hypoxia (<0.5% O_2_) suppresses energy-consuming processes in the cells such as translation and disulfide bond formation, causing protein misfolding and activating the unfolded protein response (UPR). The UPR inhibits global protein synthesis while selectively translating mRNAs to maintain endoplasmic reticulum homeostasis and promote hypoxia tolerance. Furthermore, under severe hypoxia, the ataxia–telangiectasia mutated (ATM) gene activates and DNA repair pathways are downregulated, decreasing RAD_51_ expression and impairing homologous recombination. Reoxygenation after extreme hypoxia causes DNA damage and genomic instability, increasing mutation rates and metastatic potential. Severe hypoxia also hinders the repair of G1-associated DNA double-strand breaks in irradiated cells, increasing genomic instability [2]. 

Cell survival strategies under severe hypoxia include suppressing apoptosis, initiating angiogenesis (the formation of new but often abnormal blood vessels) and erythropoiesis (the formation of red blood cells), and shifting cell metabolism from oxidative phosphorylation to glycolysis [3]. This leads to increased tumor proliferation and local invasiveness. Additionally, in the absence of oxygen, tumor cells influence immune responses and initiate rapid DNA damage repair mechanisms to maintain growth and survival, ultimately becoming resistant to radiation and chemotherapy [4,5]. 

The unique microenvironment of hypoxic tumors represents an opportunity for targeted therapy through the development of bioreductive drugs (BDs), named later hypoxia-activated prodrugs (HAPs). They are prodrugs that undergo biotransformation to cytotoxic compounds under conditions of low oxygen tension and in the presence of high levels of specific reductases [6,7]. This strategy, which originated in the early 1970s [8], allows the selective killing of cancer cells while exhibiting minimal or no toxicity to normal cells and well-oxygenated cancer cells. 

Currently, four distinct chemical entities are known to selectively target hypoxic cells: nitro(hetero)cyclic compounds, aromatic N-oxides, aliphatic N-oxides, quinones, and transition metal complexes [9]. Among them, nitroaryl- and nitroheteroaryl-based compounds show immense potential. These compounds act as bioreductive components within the chemical architecture of bioactive molecules and as triggers capable of inducing the release of the chemotherapeutic agent within hypoxic tumor regions [10]. 

**Figure 1:**
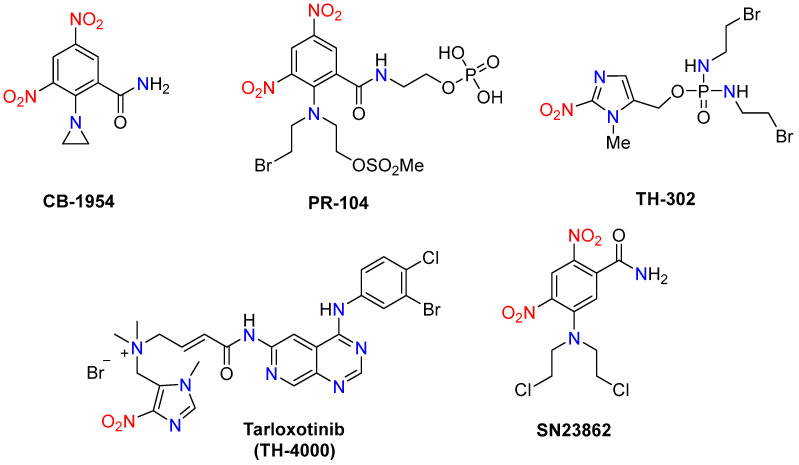
Chemical structures of some hypoxia-activated nitro(het)aromatic prodrugs.

The structures of several bioreductive agents containing nitro groups, which have been evaluated in clinical trials, are presented in Figure 1.

Despite the promising results from clinical trials of the HAPs, none have yet been commercialized. The failure in clinical trials is partly due to the following reasons: insufficient selectivity and efficacy for hypoxic tumor cells over normoxic healthy cells and potential toxicity; the highly heterogeneous hypoxic tumor environments; pharmacokinetics and stability issues etc. [11]. For example, TH-302 (evofosfamide, (1-methyl-2-nitro-1*H*-imidazol-5-yl)methyl *N*,*N*′-bis(2-bromoethyl)phosphorodiamidate), a widely studied HAP, showed limited clinical benefits in a phase I trial for advanced solid tumors, due to its insufficient selectivity for hypoxic regions and associated skin and mucosal toxicity [12]. Skin and mucosal toxicity, along with bone marrow suppression, were the most common toxicities observed when evaluating the therapeutic potential of TH-302 combined with gemcitabine for pancreatic cancer [13]. In the phase III multicenter clinical trial (TH CR-406/SARC021), 640 patients with soft tissue sarcoma were enrolled to assess the efficacy of combining TH-302 (300 mg/m²) with doxorubicin (75 mg/m²). The results showed that this combination did not improve overall survival compared to doxorubicin alone [14]. Similarly, the prodrug PR-104 (2-({2-[(2-bromoethyl)[2-(methanesulfonyloxy)-ethyl]amino]-3,5-dinitrophenyl}formami-do)etho-xy]phosphonic acid), another HAP, showed inadequate efficacy and raised safety concerns in phase II trials for relapsed or refractory acute myeloid leukemia. The most frequent treatment-related grade 3/4 adverse events were myelosuppression (anemia 62%, neutropenia 50%, thrombocytopenia 46%), febrile neutropenia (40%), infections (24%), and enterocolitis (14%) [15]. The combination of PR-104 with chemotherapeutics such as gemcitabine or docetaxel in advanced solid tumors was discontinued due to dose-limiting thrombocytopenia [16]. This necessitates the development of new nitroaryl- and/or nitroheteroaryl-based HAPs with enhanced specificity for hypoxic cells, minimized off-target effects. Additionally, improvements in the pharmacokinetic properties of HAPs via chemical modifications or formulation strategies are required.

In this review, we explore recent advancements in the design and synthesis of nitro(het)aromatic bioreductive agents, specifically focusing on their developments from 2018 to 2024. We first highlight their potential as gene-directed enzyme prodrugs that undergo controlled conversion into cytotoxic agents within target cells. Additionally, we examine the progress made in developing bioreductive-activated prodrug conjugates (BAPCs), which contain nitro(het)aromatic moieties (triggers) that activate drug molecules in the low-oxygen environment of tumors. The ability of nitro(het)aromatic compounds to undergo bioreductive activation under hypoxic conditions makes them highly effective for non-invasive tumor visualization and monitoring. We also discuss recent research on nitroaromatic compounds as tools for the fluorescent imaging of hypoxic tumors. We hope this review will aid researchers in designing new nitro(het)aromatic structures for treating and/or fluorescently imaging hypoxic tumors, paving the way for improved cancer diagnostics and treatment monitoring.

## 2. Nitro(het)aromatic Bioreductive Agents for Use in Gene-Directed Enzyme Prodrug Therapy

**Figure 2:**
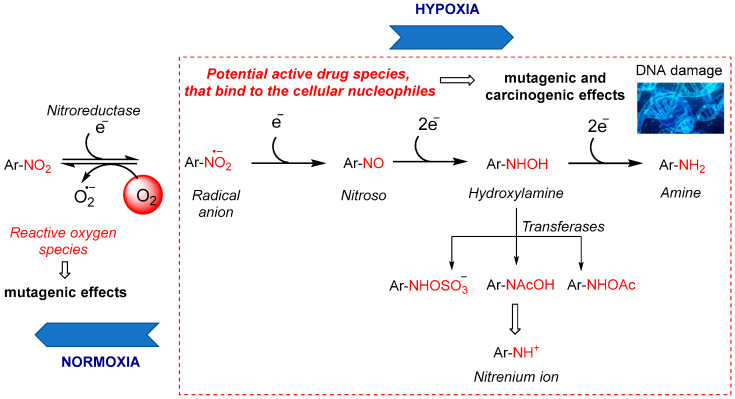
Bioreduction of nitroaryl or heteroaryl compounds under hypoxic conditions.

The mechanism by which nitro compounds localize within cells under hypoxic conditions involves multiple stages (Figure 2). In the initial and most crucial step, cellular nitroreductase enzymes reduce the nitro group of the prodrug molecule to a nitro anion radical, a short-lived species, especially in an aqueous medium. Under normal oxygen levels (normoxia), the NO_2_^.−^ radical is quickly oxidized back to the original NO_2_ group (re-oxygenated), producing superoxide anions (O_2_^.−^) in the process [17].

In the absence of oxygen, the prodrug radical anion undergoes stepwise reduction to nitroso, hydroxylamine, and amine (Figure 2). These reactive intermediates bind to cellular macromolecules such as DNA, proteins, and glutathione, thereby persisting in oxygen-deprived tissues and exhibiting mutagenic and carcinogenic effects [18]. The toxicity of nitro compounds, both desired and undesired, is linked to each of these intermediates. Hydroxylamine derivatives can cause methemoglobinemia, while nitro radical anions, nitroso derivatives, and esterified hydroxylamines (e.g., sulfo derivatives) promote mutagenic and carcinogenic effects. Additionally, superoxide anions, hydrogen peroxide, and hydroxyl radicals generated during the reduction process can also have mutagenic effects [18]. For example, the water-soluble phosphate prodrug PR-104 (Figure 1) is rapidly hydrolyzed by phosphatases in vivo to the less soluble alcohol metabolite. The latter is sufficiently lipophilic to penetrate through multiple layers of tumor cells and to reach the hypoxic target cells. There, a nitro group in the para position to the mustard residue of the prodrug undergoes reduction to hydroxylamine and an amine group, respectively. These two cytotoxic metabolites act as DNA interstrand cross-linking agents, able to diffuse locally and kill neighboring cells [19]. The bioreductive metabolic pathways of the other nitro(het)aromatic prodrugs, shown in Figure 1, are detailed in [8].

One of the most significant applications of nitro(het)aromatic compounds, in combination with enzymes from the nitroreductase group, is gene-directed enzyme prodrug therapy (GDEPT), widely explored in chemotherapy [20,21,22,23]. This therapy is a variation of direct enzyme prodrug therapy (DEPT). In DEPT, an exogenous converting enzyme is delivered to the tumor cell, rendering the cell sensitive to the administered prodrug. The advantage of using exogenous enzymes lies in their ability to activate substances that are inert to human enzymes, thereby minimizing off-target effects. However, the challenge lies in delivering these enzymes specifically to tumor cells.

**Figure 3:**
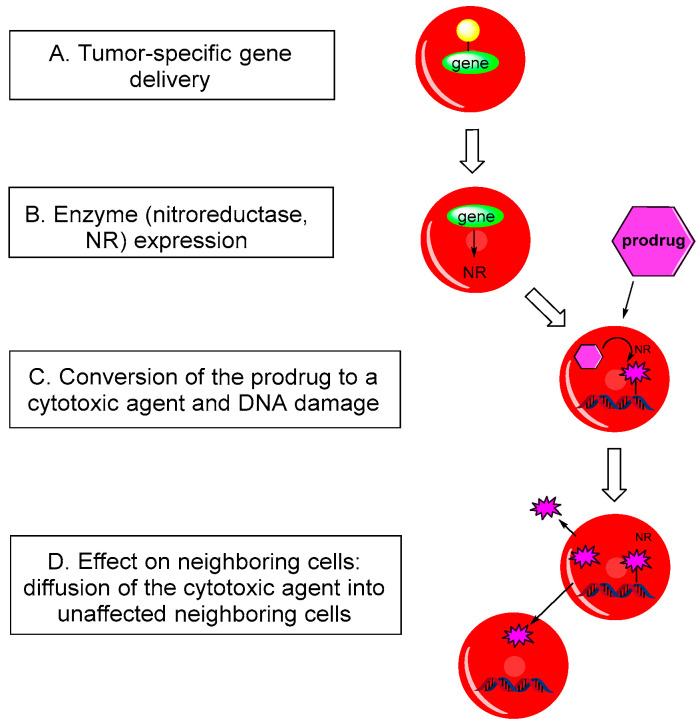
Principle of operation of GDEPT.

There are two primary approaches to enzyme prodrug therapy. The first approach involves directly delivering the enzyme linked to a tumor-targeting molecule, ensuring the enzyme reaches the cancer cells. The second approach, GDEPT, involves a more indirect strategy. GDEPT utilizes gene therapy techniques to introduce genes encoding the prodrug-activating enzyme specifically into the tumor cells [20,23]. Once inside the tumor cells, these genes are expressed, producing the enzyme that can then convert the administered prodrug into its active, cytotoxic form within the tumor microenvironment. Different nitroreductases derived from bacteria such as *Escherichia coli*, *Pseudomonas pseudoalcaligenes*, and *Staphylococcus saprophyticus* have been utilized in these studies. The GDEPT approach works through three key steps: 1. Genes encoding the nitroreductase enzyme are delivered to the tumor cells (Figure 3A). This targeting can be achieved using vectors such as viruses, plasmids, or nanoparticles engineered to selectively infect or enter tumor cells. 2. Once inside the tumor cells, the genes are transcribed and translated to produce the nitroreductase enzyme (Figure 3B). 3. The administered nitro(het)aromatic prodrug is then activated by this enzyme within the tumor cells, converting it into a cytotoxic agent that induces cell death (Figure 3C).

The specificity of GDEPT allows for high concentrations of the cytotoxic agent to be generated directly within the tumor, minimizing the damage to surrounding healthy tissues. Over the past twenty years, the GDEPT approach has seen significant progress, with numerous enzyme/prodrug systems proving effective in preclinical and clinical studies. Nonetheless, considerable efforts are still required to fully harness the potential of this promising cancer treatment option [24].

Prof. Ay. M.’s research group has worked on discovering new and effective nitroreductase—prodrug combinations for use in cancer therapy. For this purpose, they characterized a new nitroreductase, Ssap-NtrB (*Staphylococcus saprophyticus* supsp. saprophyticus), in 2012 [25], synthesized various nitro functional group-containing prodrug candidates, and investigated their enzymatic and cytotoxic effects on different cancer cells.

**Figure 4:**
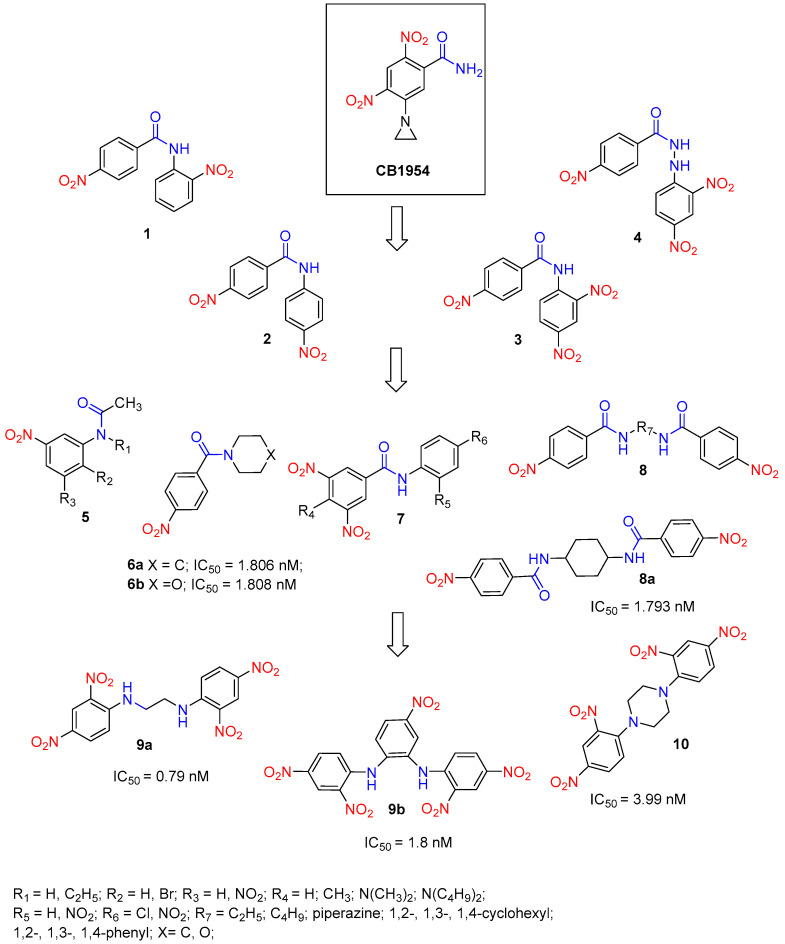
Structures of prodrug candidates **1**–**10** and a half-maximal inhibitory concentration (IC_50_) of their metabolites.

Güngör T. et al. designed prodrugs **1**–**4** based on the model prodrug CB1954 and some benzamides. Their concept involved replacing the -CONH_2_ group of CB1954 with the nitro group containing -CONHAr groups (Figure 4) [26]. According to the HPLC results, all prodrugs were activated by Ssap-NtrB. Prodrugs **1** and **2** produced one metabolite each, while prodrugs **3** and **4** produced three metabolites each upon activation. Derivative **3** was significantly more active than CB1954 and SN23862, with 137- and 31-fold higher activity for Ssap-NtrB, respectively. Among all the prodrug metabolites following Ssap-NtrB reduction, *N*-(2,4-dinitrophenyl)-4-nitrobenzamide **3** was notably effective and toxic to PC3 cells, comparable to CB1954. Kinetic parameters, molecular docking, and the HPLC results also indicated that prodrug 3 interacts more favorably with Ssap-NtrB than prodrugs 1, 2, and 4, or the known cancer prodrugs CB1954 and SN23862. This makes prodrug 3 a promising candidate for NTR-based cancer therapy.

The metabolites of prodrugs **6a** and **6b** exhibited IC_50_ values of 1.806 nM and 1.808 nM, respectively [27]. The metabolite of prodrug **8a** demonstrated an IC_50_ value of 1.793 nM, comparable to CB1954. The common structural feature of the most active nitrobenzamide compounds (**6a**, **6b**, and **8a**) includes a nitro group in p-position on the phenyl core relative to the amide group and the presence of nitrogen-containing heterocyclic systems such as piperidine (**6a**), morpholine (**6b**), or a saturated 1,4-cyclohexyl moiety (**8a**). The compounds with two phenyl nuclei and one amide group exhibited the highest toxicity, followed by bis-benzamides. The toxicity of the tested benzamides can be ranked in the following order: **6** < **5** < **8** < **7**. As a result of theoretical and biological studies, combinations of **6a**, **6b,** and **8a** with Ssap-NtrB can be suggested as potential prodrugs–enzyme combinations at nitroreductase-based cancer therapy, compared with the CB1954–NfsB combination.

Further in this direction, Tokay et al. present the synthesis of *N*-(substituted)-2,4-dinitroaniline derivatives, in particular symmetrical bis(2,4-dinitrophenyl)diamine derivatives **9** and *N*-(5-morpholino-2,4-dinitro phenyl)alkanamides **10**. These aromatic secondary amines were derived from 2,4-dinitro-1-chlorobenzene and various aliphatic, alicyclic, aromatic, or heterocyclic diamino derivatives utilizing Et_3_N or NaH as a base in DMF solvent at room temperature or 60–70 °C via the SNAr reaction mechanism [28]. The design of these compounds was based on model bioreductive dinitroaniline prodrugs such as CB1954, SN23862, and PR-104A. The cytotoxic effects of prodrug candidates were assessed using the MTT assay on human hepatoma cells (Hep3B), prostate cancer cells (PC-3), and human umbilical vein endothelial cells (HUVEC) as healthy controls. The compounds with minimal toxicity were further investigated to evaluate their potential as prodrug candidates. Biochemical analyses were conducted to examine the reduction profiles and kinetics of prodrug–Ssap-NtrB combinations. Subsequently, selected prodrug–Ssap-NtrB combinations were applied to prostate cancer cells to assess their toxicity. The combined results from theoretical, in vitro cytotoxic, and biochemical studies indicate that prodrug/enzyme combinations such as **9a**—Ssap-NtrB, **9b**—Ssap-NtrB, and **10**—Ssap-NtrB hold promise as potential candidates for nitroreductase (Ntr)-based prostate cancer therapy.

**Figure 5:**
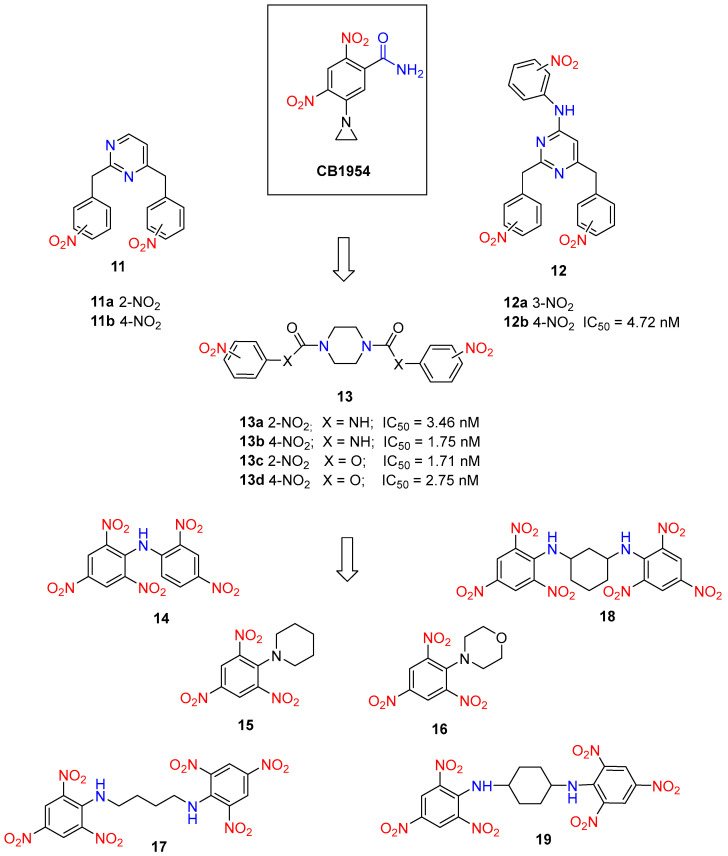
Structures of prodrug candidates **11**–**19** and a half-maximal inhibitory concentration (IC_50_) of their metabolites.

Building on their previous works, Güngör and colleagues synthesized a series of *N*-heterocyclic nitro prodrugs (**11**–**13**, Figure 5) containing pyrimidine, triazine, and piperazine rings. Nitro-containing triazine derivatives **11** and **12** are synthesized from cyanuric chloride and the amines via a nucleophilic substitution reaction. The process involves refluxing cyanuric chloride and aromatic amines in acetic acid for varying reaction times (15 min to 24 h), followed by purification through crystallization with isopropyl alcohol. For the synthesis of urea derivatives of nitrophenyls and piperazine **13a**–**b**, a Curtius rearrangement is applied. This involves first reacting nitrobenzoyl chlorides with sodium azide to obtain nitrobenzoyl azide derivatives. Subsequently, at the reflux temperature of toluene, nitrophenyl isocyanate forms as an unstable intermediate, which then reacts with piperazine, yielding the desired products with high efficiency (78–96%). Prodrugs **13c**–**d**, the carbamate derivatives of nitrophenyls and piperazine, were synthesized using Rivett and Wilshire’s method. The synthesis begins with 1,4-bis(chlorocarbonyl)piperazine, obtained from the reaction of piperazine with phosgene. This intermediate is then reacted with nitrophenols (2-nitro, 3-nitro, and 4-nitro) in DMF at room temperature using NaH as the base [29]. 

The compounds displayed varying cytotoxic profiles. For example, the pyrimidine derivative **11b** and the triazine derivative **12a** emerged as promising drug candidates for prostate cancer with IC_50_ values of 54.75 µM and 48.9 µM, respectively. Compounds **12b**, **13a**–**c** were identified as prodrug candidates due to their non-toxic properties across three different cell models. The prodrug capabilities of these selected compounds were assessed using the SRB assay in combination with Ssap-NtrB. SRB screening results indicated that the metabolites of all selected non-toxic compounds exhibited significant cytotoxicity, with IC_50_ values ranging from 1.71 to 4.72 nM, against prostate cancer cells. Among the tested compounds, piperazine derivatives **13b** and **13c** showed particularly notable toxic effects, with IC_50_ values of 1.75 nM and 1.71 nM, respectively, against PC3 cells, comparable to the standard prodrug CB1954 (IC_50_ = 1.71 nM) [29]. 

Based on enzymatic studies, prodrugs **15** and **18** (Figure 5) demonstrated the highest activity with Ssap-NtrB during short incubation periods, and their metabolite profiles were examined in detail over time. Similarly, **15** and **18** showed efficient enzymatic reduction by Ssap-NtrB. In contrast, **14** and **15** exhibited no interaction with nitroreductase within the limited timeframe, and the interaction levels for **17** and **19** were found to be insufficient. Furthermore, kinetic studies revealed that the catalytic efficiencies of the Ssap-NtrB/TNA1, Ssap-NtrB—**15**, and Ssap-NtrB—morpholine analog **16** combinations were 61, 28, and 20 times higher, respectively, than that of *E. coli* NfsB-CB1954 [30]. 

Although brief, this review highlights recent advances in the synthesis and structural modifications of nitroaromatic prodrugs, with potential applications in suicide gene therapy. It is hoped that these insights will pave the way for the design and synthesis of novel bioreductive agents for GDEPT.

## 3. Bioreductive-Activated Prodrugs Conjugates (BAPCs)

**Figure 6:**
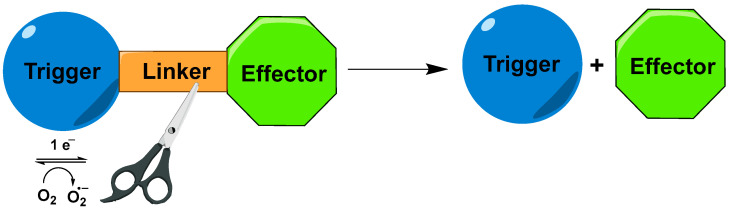
Reductive fragmentation of hypoxia-activated cytotoxins.

Another strategy for cancer therapy that targets hypoxia involves using hypoxia-activated prodrugs (triggers), which preferentially release chemotherapeutic agents (effectors) within hypoxic tumor regions. Under oxygen-poor conditions, the functional groups in these prodrugs (such as nitrophenyl, nitrobenzyl, or nitroheteroaryl triggers) are selectively reduced by reductases to electron-donating groups such as amine (-NH_2_) or hydroxylamine (-NHOH), resulting in a dramatic change in the electron density of the aromatic moiety. The released electrons cause fragmentation of the linker and release the cytotoxic agent into the tumor, while leaving non-hypoxic cells undamaged (Figure 6) [6]. This approach can enhance therapeutic effectiveness compared to conventional chemotherapeutic treatments by concentrating the drugs within hypoxic tumor environments. At the same time, it reduces the side effects and toxicity associated with the systemic distribution of traditional drugs on normoxic cells [31].

The trigger’s role can be fulfilled by any of the above-mentioned bioreductive units, such as nitrophenyl, nitrobenzyl, nitro heteroaryl, azo compounds, quinones, or oxides [32,33,34]. The effector unit needs to have high cytotoxicity and effectiveness against multiple cancer types [35]. Commonly used effectors include drugs such as Doxorubicin (DOX), Camptothecin, and Paclitaxel (PTX) [36,37]. The linker, which connects the trigger to the chemotherapeutic agent, ensures stability in the bloodstream, while allowing for efficient release in the tumor environment. Preferred linkers are ether, ester, or carbamate subunits, due to their biocompatibility, stability in the bloodstream, and sensitivity to specific enzymes or acidic conditions, which are more prevalent in tumor tissues than in normal tissues [38].

**Figure 7:**
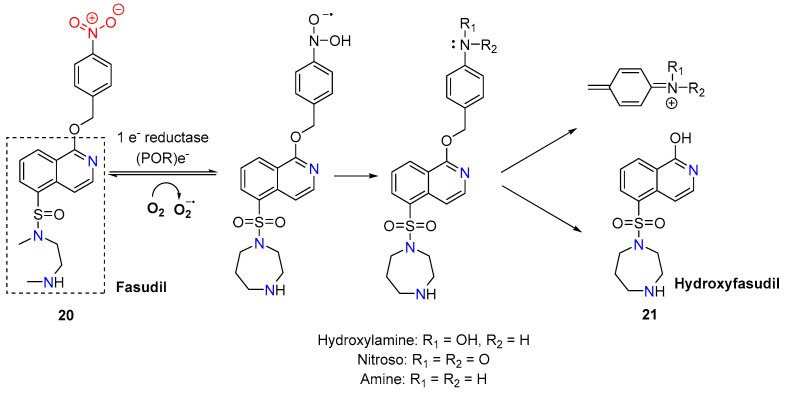
Scheme of the bioreduction process of conjugate **20** to hydroxyfasudil **21**.

By masking Fasudil’s active site with a bioreductive 4-nitrobenzyl group, Al-Kilal et al. [39] synthesized the conjugate **20** (Figure 7). Under normoxic conditions, the conjugate exhibited significantly reduced antineoplastic activity (IC_50_ = 6.8 μM) compared to the parent compound (IC_50_ = 0.48 μM). However, under severe hypoxia, the nitro group is reduced to form an electron-donating substituent, which induces fragmentation and ejects the hydroxyfasudil **21.** This process significantly enhanced the antiproliferative effect on disease-afflicted pulmonary arterial smooth muscle cells and pulmonary arterial endothelial cells (IC_50_ = 0.40 μM).

**Figure 8:**
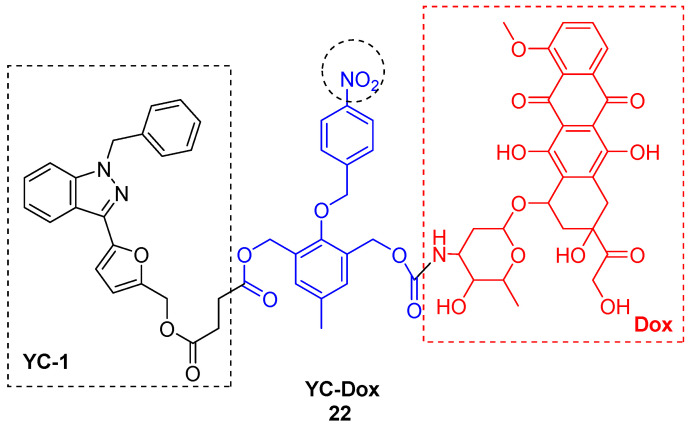
Chemical structure of bioreductive-activated prodrug conjugate **YC-Dox 22**.

Nitrobenzyl trigger was used in the construction of hypoxia-activated prodrug YC-Dox **22** (Figure 8) [31]. This prodrug is capable of specifically releasing the chemotherapeutic agent Dox and the HIF-1α (hypoxia-inducible factor-1α) inhibitor YC-1 hemisuccinate (3-(5′-hydroxymethyl-2′-furyl)-1-benzylindazole) in response to hypoxia.

It is well known that low oxygen levels in hypoxic tumor tissues lead to the accumulation of HIF-1α. This protein plays a crucial role in the adaptive response of cancer cells to hypoxia by regulating various cellular functions [40]. YC-1 is capable of blocking HIF-1α expression and consequently inhibiting the activity of HIF-1 as a transcription factor in hypoxic cancer cells, leading to the suppression of tumor growth. In addition, YC1 exhibits antiproliferative effects [41,42]. The release of Dox and YC-1 from the prodrug YC-Dox in response to hypoxia results in substantial synergistic potency against hypoxic cancer cells and remarkable cytotoxic selectivity, being more than eight times greater compared to normoxic healthy cells. In vivo experiments demonstrate that this prodrug can selectively target hypoxic cancer cells while avoiding unintended effects on normal cells. This selective targeting results in enhanced therapeutic efficacy for tumor treatment and reduced adverse effects on normal tissues.

**Figure 9:**
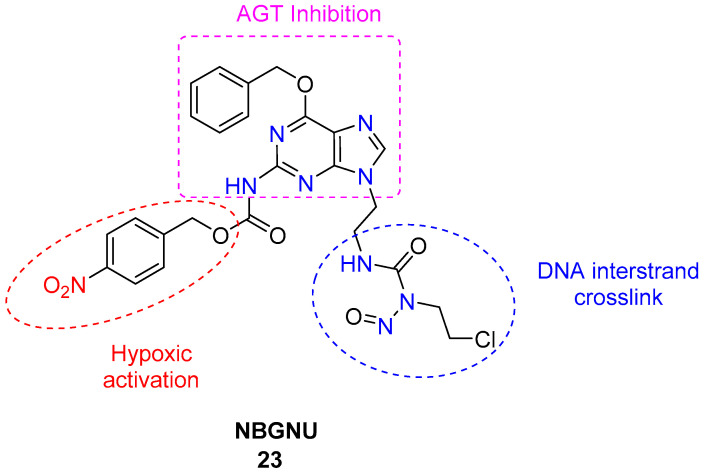
Chemical structure of bioreductive-activated prodrug conjugate **NBGNU 23.**

Ce, Y et al. reported the synthesis of the hypoxia-activated prodrug, *N*-(2-chloroethyl)-*N*-2-(2-(4-nitrobenzylcarbamate)-O^6^-benzyl-9-guanine)ethyl-*N*-nitrosourea (NBGNU), **23** (Figure 9) [43]. The molecule integrates the chloroethylnitrosourea (CENU) pharmacophore to induce DNA interstrand cross-links and an O^6^-benzylguanine analog moiety (angiotensinogen (AGT) inhibitor) masked by a 4-nitrobenzylcarbamate group to induce hypoxia-activated inhibition of O^6^-alkylguanine-DNA alkyltransferase. Its anticancer effectiveness was assessed through in vitro experiments. The prodrug demonstrated promising antitumor efficacy and hypoxic selectivity due to the incorporation of an AGT inhibitor and hypoxia-activated pharmacophores into the side chain of the CENU moiety. The activity of **23** against AGT-expressing human glioma SF763 cells under hypoxic conditions (IC_50_ = 126 μM) was significantly higher than under normoxic conditions (IC_50_ = 580 μM). This indicates that NBGNU selectively undergoes reduction under hypoxic conditions, leading to the unmasking of the 2-amino group of guanine, the release of O^6^-BG analogs, and the effective inhibition of AGT. However, to further enhance its hypoxic selectivity and chemotherapeutic efficacy, improvements are needed to reduce normoxia activation and increase water solubility.

**Figure 10:**
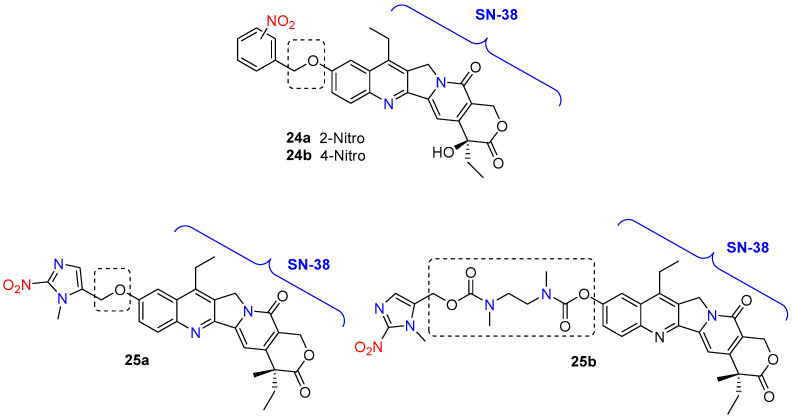
Chemical structure of bioreductive-activated prodrugs conjugates **24a**,**b** and **25a**,**b**.

The 2-nitrobenzyl- and 4-nitrobenzyl-SN-38 analogs **24a**,**b** (Figure 10), as discussed by Liang et al. [44], embody a key feature of hypoxia-activated prodrugs: they are significantly less potent than their active metabolite. Initial cell viability assays showed that these analogs were considerably less cytotoxic than SN-38 against human leukemia K562 cells, with 2-nitrobenzyl-SN-38 **24a** and 4-nitrobenzyl-SN-38 **24b** displaying 8-fold and 19-fold lower cytotoxicity, respectively. Furthermore, in a topoisomerase I assay, the 4-nitrobenzyl analog at the C-10 position of SN-38 inhibited the enzyme’s ability to relax supercoiled pBR322 DNA at concentrations similar to the clinically approved SN-38. Although the reduction potentials of these compounds were lower than those of other known HAPs and partially reversible, they demonstrated potential as hypoxia-targeted therapeutics. The study concluded that the next generation of SN-38-HAPs should incorporate bulkier nitroaromatic groups to further reduce cytotoxicity and use triggers with higher reduction potentials to align with the range of cellular reductases (−450 to −300 mV).

The 2-nitroimidazole fragment is a widely used trigger in the fragmentation concept, due to its good hydrophilicity and its relatively high one-electron reduction potential, well within the range of various reductase enzymes, and high selectivity for hypoxic conditions. Furthermore, there is a significant body of clinical and preclinical data supporting the efficacy and safety of 2-nitroimidazole-based prodrugs. This accumulated evidence provides a strong foundation for their continued use and further development.

Choi and co-workers [45] conjugate the camptothecin derivate SN-38 with 1-methyl-2-nitro-1*H*-imidazole-5-yl fragment using two different linkers—an ether linkage **25a** and carbamate functionality **25b** (Figure 10). The ether analog **25a** had moderate hypoxia selectivity and more toxicity compared with TH-302. The different linkers in the structures of the two derivatives likely account for the significant differences in their hypoxia selectivity and toxicity. Compound **25a** demonstrated ten times higher toxicity against the human lung cancer cell line H460 and the human colon cancer cell line HT29 compared to the control hypoxia-activated nitroimidazole prodrug TH-302. Furthermore, **25a** exhibited lower toxicity than SN-38 under normoxic conditions. However, both the hypoxic selectivity and toxicity of **25a** were lower compared to those of compound **25b**. Despite this, the ether-linked compound 3a is considered a promising hypoxia-selective antitumor agent.

**Figure 11:**
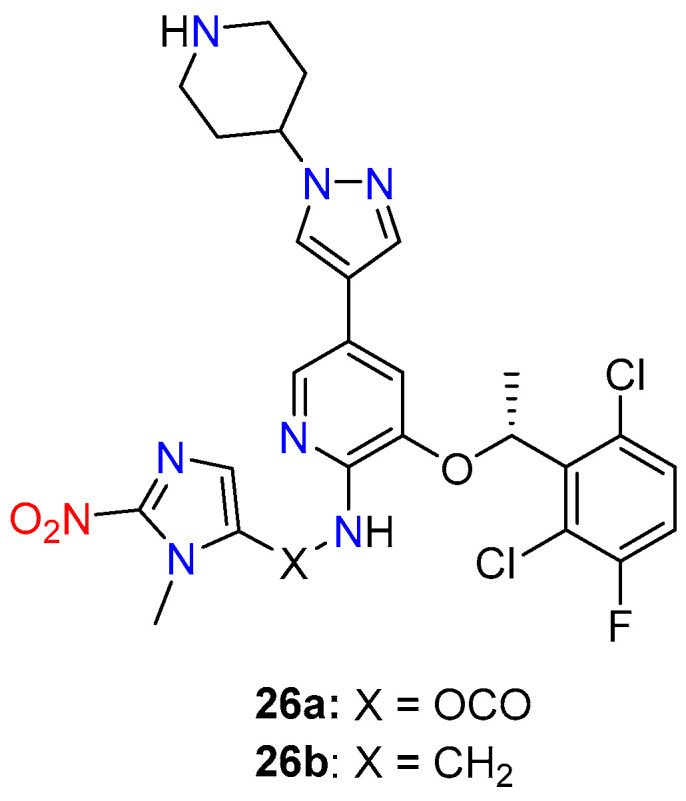
Chemical structure of bioreductive-activated prodrugs conjugates **26a**,**b**.

Bielec, B. and colleagues [46] developed the first crizotinib prodrugs **26a**,**b** (Figure 11) aimed at reducing severe adverse effects and enhancing anticancer activity. The design of these prodrugs involves a hypoxia-activatable, self-immolative 2- nitroimidazole trigger moiety at a key tyrosine kinase binding site of crizotinib, which significantly reduces its affinity for the catalytic pockets of the target kinases c-MET and ALK. Two different prodrug derivatives were synthesized: one with the trigger moiety coupled via carbamoylation (**26a**) and the other via alkylation (**26b**) of the 2-aminopyridine moiety of crizotinib. Prodrug **26a** demonstrated high stability in serum, a crucial requirement for successful prodrug development, and effectively inhibited c-MET phosphorylation and cell proliferation in tumor tissues in vivo following intravenous application. Overall, the data suggest that prodrug **26a** is a promising candidate for further (pre)clinical development as a novel tyrosine kinase inhibitor with improved tumor-specific properties.

**Figure 12:**
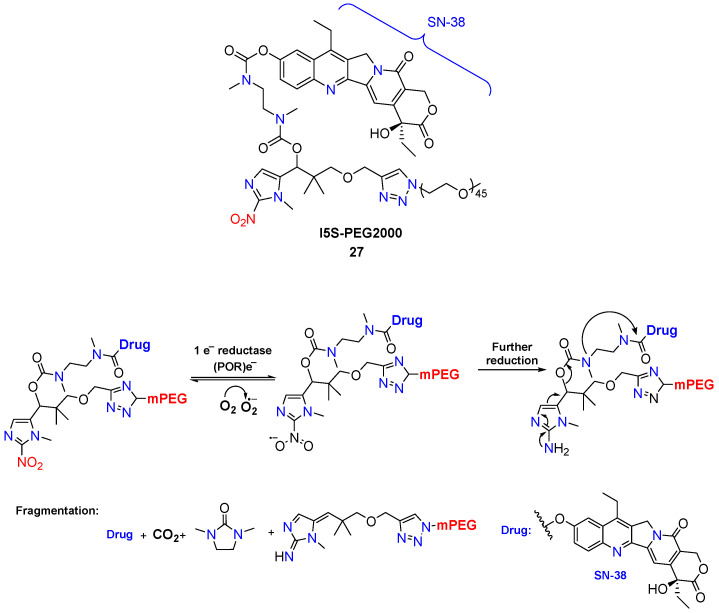
Scheme of the bioreduction process of conjugate **27** to the camptothecin derivative SN-38.

In another study, a hypoxia-activated camptothecin derivative embodies a multifunctional bioreductive linker based on 1-methyl-2-nitroimidazole. The incorporation of a PEG chain in the linker increased the water solubility of the SN-38- prodrug **27** and ensured stability under physiological conditions [47]. When conjugated with SN-38, this linker demonstrated the capability to efficiently release the drug through a two-step process: reductive activation of the 2-nitroimidazole, followed by spontaneous degradation of the linker via 1,6-elimination and cyclization-elimination, ultimately resulting in drug molecule release (Figure 12).

**Figure 13:**
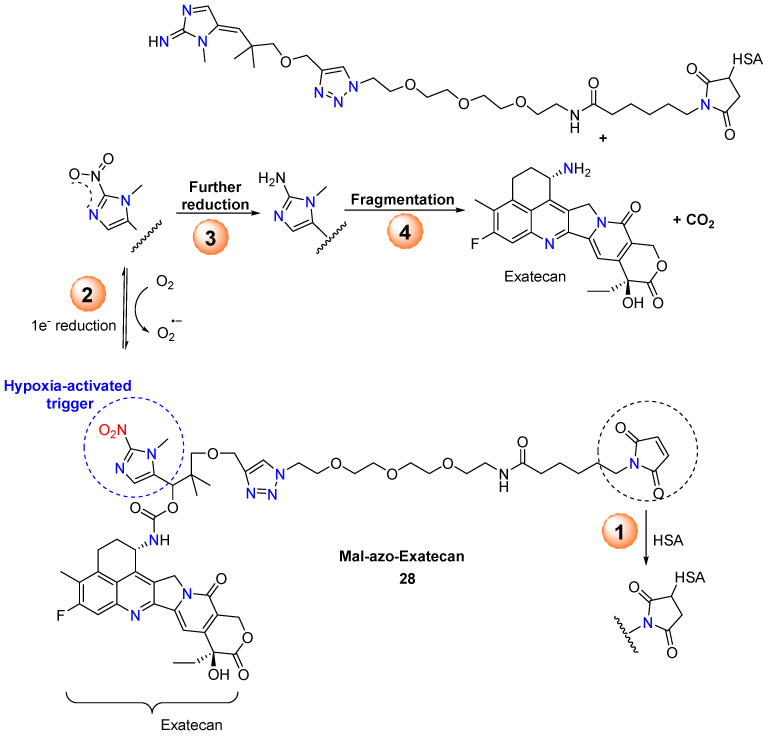
Scheme on the bioreduction process of conjugate **28** to Exatecan.

Encouraged by the great properties of the multifunctional linger-containing 1-methyl-2-nitroimidazole trigger unit [47], Chang et al. reported the synthesis and biological evaluation of the hypoxia-activated albumin-binding prodrug Mal-azo-Exatecan **28** (Figure 13) [48]. The 5-position branched linker of 1-methyl-2-nitro-5-hydroxymethylimidazole served as a hypoxic cleavage trigger, linking the camptothecin analog Exatecan via a carbamate bond. After intravenous administration, the side-chain maleimide rapidly binds to human serum albumin (HSA). The HSA-azo-Exatecan carrier system accumulates in tumor tissue through the enhanced permeability and retention effect, as well as the interaction with the albumin receptor gp60. In the hypoxic tumor environment, Exatecan is released, triggered by nitroreductase. The nitroimidazole trigger has high plasma stability and does not cause the chemotherapeutic agent release from HSA-azo-Exatecan during circulation in vivo, avoiding systemic side drug effects.

**Figure 14:**
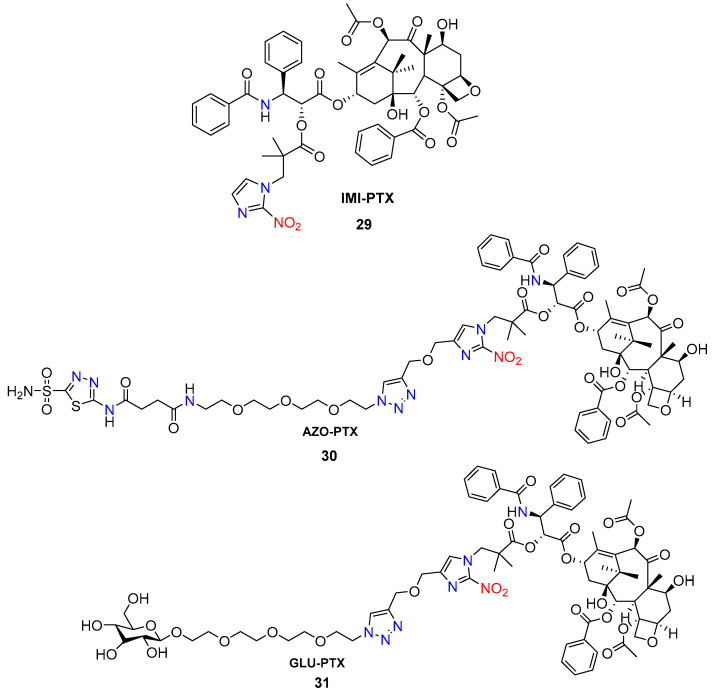
Chemical structure of bioreductive-activated prodrugs conjugates **29**–**31**.

Zhang and co-workers [49] reported the synthesis of hypoxia-activated paclitaxel (PTX) prodrugs IMI-PXT **29** based on hypoxia-sensitive 2-nitroimidazole moiety. The 2-nitroimidazole unit at the N-1 position is connected to 2′-OH of PTX via an ester bond with pivalic acid for easier drug release. The GLU-PTX **30** and AZO-PTX **31** prodrugs (Figure 14) contain glucose and acetazolamide as targeting ligands.

In contrast to the 2-nitroimidazole unit, 2-nitrothiophene and 2-nitrofuran triggers in hypoxia-activated prodrugs exhibit less favorable stability, reactivity, and toxicity profiles, rendering them less attractive for the development of HAPs. 

**Figure 15:**
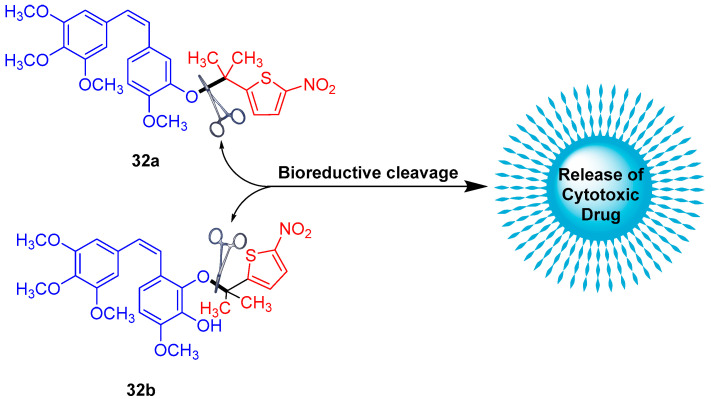
Chemical structure of gem-dimethyl prodrugs **32a**,**b**.

Winn et al. reported the synthesis of the scombretastatin A-1 (CA1) and combretastatin A-4 (CA4) prodrug conjugates **32a**,**b** (Figure 15) [50]. The most active compounds in the series were the gem-dimethyl prodrugs of CA1 (**32a**) and CA4 (**32b**)**,** exhibiting hypoxia cytotoxicity ratios of 12.5 and 41.5, respectively. This high selectivity is attributed to the gem-dimethyl CA4-BAPC’s enhanced resistance to cleavage in oxygenated environments, allowing the parent anticancer agent (CA4) to be released selectively under hypoxic conditions.

## 4. Nitroaromatic Compounds as Fluorescent Probes for Hypoxia Detection and Imagining

Conventional methods for the in vivo imaging and detection of solid tumors are typically applied only in advanced stages of cancer. Hypoxia imaging, however, offers a promising alternative for earlier cancer diagnosis, enabling tumor visualization with a diameter as small as 350 μm [51]. The major techniques for hypoxia measurements in tumors include immunohistochemical staining, oxygen electrodes, DNA strand breaks, polarographic needle electrodes, magnetic resonance imaging, positron emission tomography, single-photon emission computed tomography (SPECT), and fluorescence imaging. In recent decades, fluorescence imaging has emerged as one of the most advanced methods for quantifying hypoxia. It offers several advantages, such as non-invasiveness, higher sensitivity, real-time monitoring in living systems, absence of ionizing radiation, low toxicity, simple operation, and low cost [51,52,53,54,55]. 

Due to the relatively easy synthesis, well-predictable and highly selective fluorescent sensing output, the nitro aromatic molecules have become the most attractive approach in the design of fluorescent probes for hypoxia conditions [56,57,58,59]. Even commercially available options for in vivo studies of hypoxia, such as pimonidazole (alpha-((2-Nitroimidazol-1-yl)methyl)-1-piperidineethanol), are based on nitro-containing compounds [60]. 

**Figure 16:**
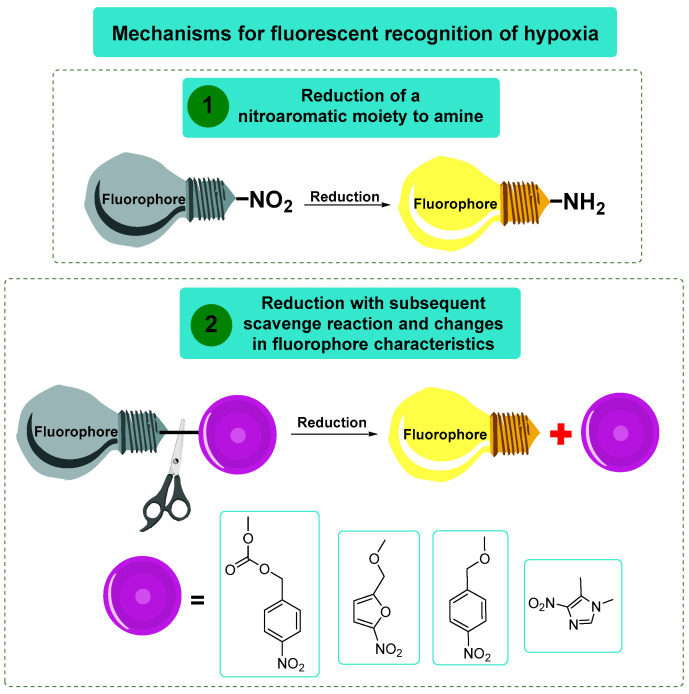
The basic approaches for designing fluorescent hypoxia-activated probes.

To date, the nitro aromatic compounds were used as a platform for fluorescent recognition of hypoxia according to two major mechanisms, and both were based on the bioreduction of the nitro aromatic system. The first one referred to the selective labeling of hypoxic cells due to the reduction of a nitroaromatic moiety in the fluorophore architecture to amine. The main concept here lies in the fact that the nitro group is well known as a fluorescence quencher in aromatic systems, but after the bioreduction of this nitro group in hypoxic conditions, the aromatic systems become fluorescent. The second mechanism was based on the reduction of a fluorescent probe containing 4-nitrobenzyl formiatic, 4-nitrobenzylic, or similar heterocyclic fragments in hypoxic cells, which resulted in a scavenge reaction and alterations in former fluorescence wavelength or intensity (Figure 16).

**Figure 17:**
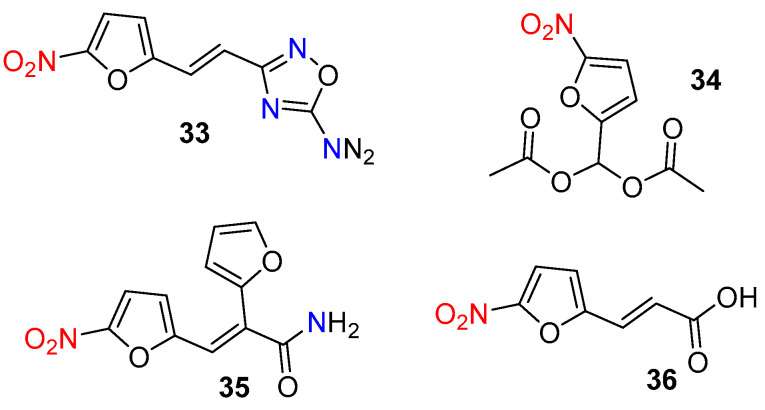
Chemical structures of hypoxia-activated fluorescent probes **33**–**36**.

The development of fluorescent probes for hypoxia imaging began in the 1980s and 1990s, focusing on the bioreduction of nitroaromatic compounds to amines. Olive and Durand were among the first to reveal the significant potential of nitroaromatic rings for hypoxia imaging [61,62]. In their reports, they demonstrated that relatively nontoxic nitrofurans **33**–**36** (Figure 17) exhibit a highly responsive fluorescent output to intracellular oxygen concentration under hypoxic conditions.

**Figure 18:**
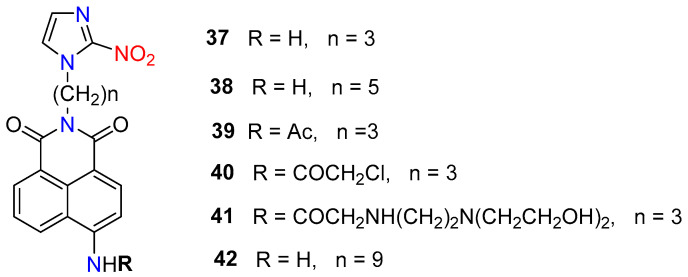
Chemical structures of hypoxia-activated 1,8-naphthalimide probes **37**–**42**.

Later, Hodgkiss et al. obtained a family of hypoxia-activated fluorescent naphthalimides (**37**–**42**) containing 2-nitroimidazole side chains (Figure 18). These molecules were nonfluorescent due to the photoinduced electron transfer (from the fluorophore-excited state to the nitroaromatic moiety), which was prevented after enzymatic bioreduction [55]. Furthermore, it is well known that the bioreduction of nitroimidazoles leads to intracellular bioreductive metabolites that react with biomolecules, thus providing a binding mechanism for the fluorescent probes to hypoxic cells. Since this report, the 2-nitroimidazole side chains have become a major tracer for hypoxic cells and remain popular to date.

**Figure 19:**
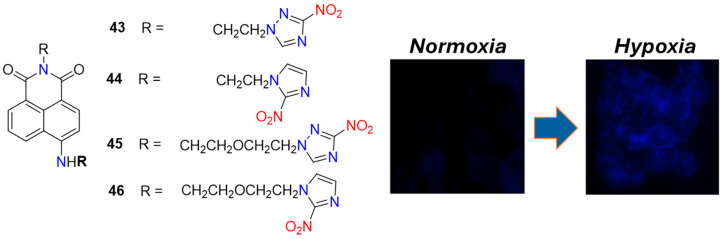
Chemical structures of hypoxia-activated 1,8-naphthalimide probes **43**–**46** and V79 cells incubated with **46** under normoxic and hypoxic conditions. Adapted with permission from [63]. Copyright (2006) Elsevier.

Due to the relatively easy synthesis and well-predicted off-on fluorescent response to intracellular oxygen concentration, numerous nitroaromatic structures have been developed for hypoxia imaging based on the two principles mentioned above: the bioreduction of a nitro group directly attached to the fluorophoric system, or the use of 2-nitroimidazole side chains. For example, Qian et al. extended the concept of Hodgkiss et al. by preparing similar naphthalimides (**43**–**46**, Figure 19) but with two 2-nitroimidazole fragments instead of one, thus reporting the first hypoxic probes containing two heterocyclic-binding side chains [63]. The main motive for the synthesis of compounds **44** and **46** was the study of the side chain effect in C4-position of the naphthalimide ring, which plays a more important role than the fluorophoric architecture itself during interaction with DNA and probe interference. The authors discovered that in V79 cells, probe **46** exhibited a higher fluorescence enhancement (FE = 20 times) compared to **44** (FE = 15 times). This difference in signal responses between the two probes was attributed to the shorter side-chain length of **44** compared to **46,** which reduces the possibility of fluorescent quenching due to intramolecular photoinduced electron transfer from the reduction products of the nitroimidazole moiety to the excited naphthalimide fluorophore.

**Figure 20:**
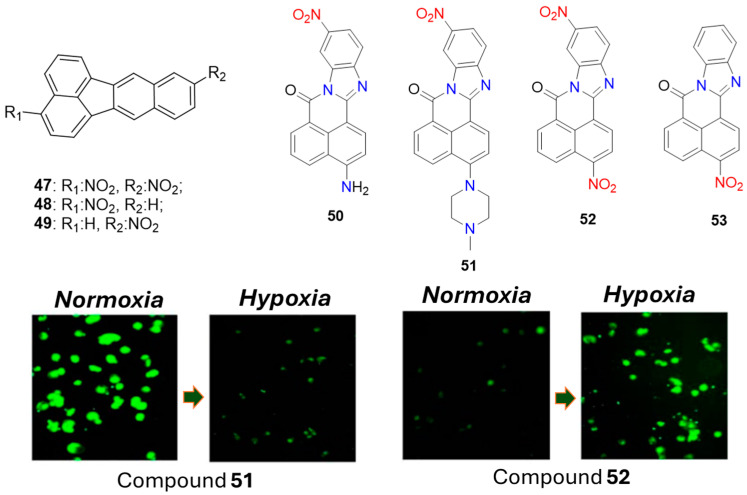
Chemical structures of hypoxia-activated 1,8-naphthalimide probes **47**–**53** and V79 cells incubated with **51** (left) and **52** (right) under normoxic and hypoxic conditions. Adapted with permission from [65]. Copyright (2008) Springer Nature.

Qian et al. were motivated by the ease of synthesis and low cost to develop a series of hypoxic probes (**47**–**53**) containing a nitro group directly incorporated into the fluorophoric scaffold (Figure 20) [64,65]. Compounds **47**–**49** demonstrated promising fluorescent responses in hypoxic V79 cells, with the hypoxic-oxic fluorescence differential reaching 6, 9, and 11 times after incubation with **47**, **48**, and **49**, respectively. However, the observed increase in fluorescence enhancement correlated with the probes’ increased water solubility. This indicates that water solubility was a general issue with these probes, as deposition outside the cells hindered future quantitative analysis.

Furthermore, with probes **50**–**53**, Qian et al. demonstrated that the NO_2_ group in fluorogenic compounds could function not only as a fluorescent quencher but also as an electron acceptor that enhances fluorescence emission. Probes **52** and **53** exhibited the usual off-on fluorescent switching upon transitioning from oxic to hypoxic conditions, showing a 12-fold fluorescence enhancement. However, the analogous probes **50** and **51**, which contained the nitro group only in the electron-accepting part of the fluorophoric system, displayed the opposite fluorescent response—a 14-fold fluorescence quenching under hypoxic conditions compared to oxic conditions. This was attributed to the internal charge transfer (ICT) nature of the fluorophore, where fluorescence appears due to the charge transfer from electron-donating amines to the nitro-containing electron-accepting part of the molecule. The bioreduction of this nitro group to an electron-rich amine destabilized the ICT state, which is crucial for strong fluorescence. Additionally, the authors found that under hypoxic conditions in V79 cells, the nitro group in the naphthalene ring was reduced preferentially over the one in the benzene ring. This selective reduction was essential for maintaining the observed fluorescent on-state of probe **52** in hypoxia.

**Figure 21:**
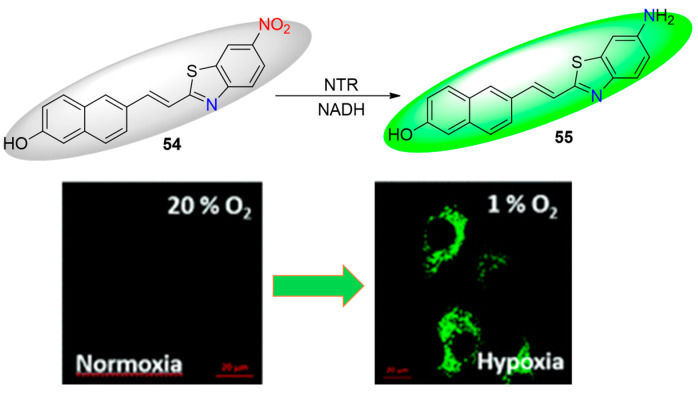
Chemical structures of hypoxia-activated probe **54** and HeLa cells incubated with **54** under normoxic and hypoxic conditions. Reproduced with permission from [66]. Copyright (2020) The Royal Society of Chemistry.

The concept of incorporating a nitro group directly into the fluorophoric system, which activates fluorescence emission due to the favored intramolecular charge transfer after bioreduction, remains relevant for designing hypoxic probes. Recently, Fan et al. reported a non-fluorescent benzothiazole probe, **54** (Figure 21) [66]. Upon reaction with nitroreductase (NTR), the nitro group in probe 54 underwent enzyme-catalyzed reduction to an amine, resulting in a strong fluorescent emission. This process demonstrated high sensitivity, with a detection limit of 48 ng/mL and a linear range of 0.5–8.0 μM for NTR. Additionally, **54** was successfully used for imaging hypoxia levels in living HeLa cells, rat tumor tissues, and zebrafish. These results, combined with the probe’s low toxicity, indicate significant potential for detecting hypoxia in solid tumors.

**Figure 22:**
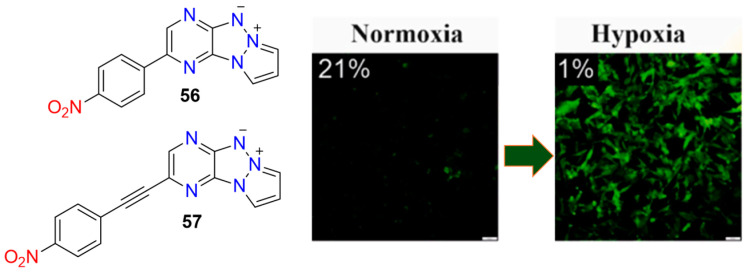
Chemical structures of hypoxia-activated 1,8-naphthalimide probes **56** and **57**, and HeLa cells incubated with **56** under normoxic and hypoxic conditions. Adapted with permission from [67]. Copyright (2021) Elsevier.

Janczy-Cempa et al. employed a similar strategy in the design of two nitro-pyrazinotriazapentalene derivatives, **56** and **57** (Figure 22), initially exhibiting weak fluorescence [67]. Upon reduction of their nitro groups by NTR, a significant fluorescence enhancement with a 15-fold increase in intensity was observed. The reduction process with NTR was selective, with linear ranges of 0–4 μg/mL and limits of detection of 18.6 ng/mL for **56** and 33.2 ng/mL for **57**, respectively. Both probes were non-toxic and successfully employed for imaging hypoxia in the A2058 cell line.

**Figure 23:**
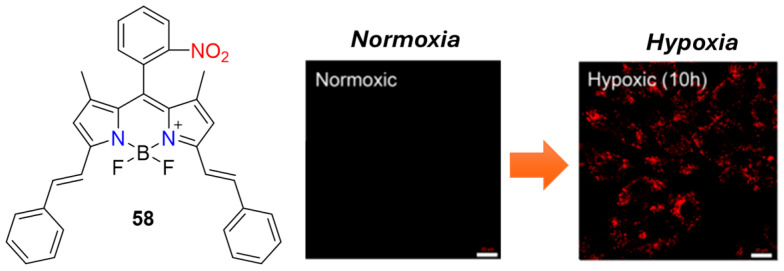
Chemical structures of hypoxia-activated probe **58** and H9c2 cells incubated with **58** under normoxic and hypoxic conditions. Adapted with permission from [68]. Copyright (2019) American Chemical Society.

The current focus on developing highly emissive fluorescent probes in the near-infrared (NIR) region is driven by NIR’s superior penetration ability in living systems, effectively minimizing interfering bio-autofluorescence. This motivation led Fan et al. to synthesize a BODIPY-based fluorescent probe, **58**, for hypoxia imaging (Figure 23) [68]. Initially non-emissive, compound **58** contained a fluorescent-quenching nitro group that could be selectively reduced by NTR to its amine form. Upon reduction, the resulting amino derivative exhibited bright fluorescence in the NIR spectrum at 713 nm. Unlike its precursor, the fluorescence of the amino derivative of **58** showed a linear increase, correlated with the NTR concentration in the range of 0.1−1.0 μg/mL. A significant 55-fold maximum fluorescence enhancement was achieved, with a calculated limit of detection (LOD) of 7.08 ng/mL. Building upon probe **58**, nanoparticles were developed and used as fluorescent probes for hypoxia imaging both in vitro (H9c2 cells) and in vivo (ischemic mice model). These nano-probes demonstrated negligible toxic effects, making them suitable for in vivo applications.

**Figure 24:**
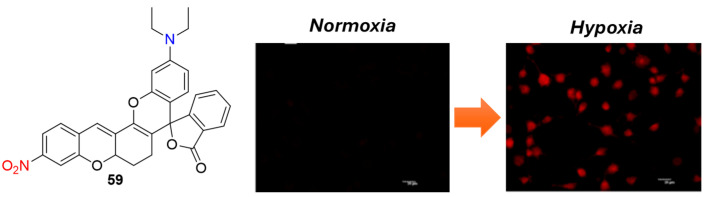
Chemical structures of hypoxia-activated probe **59** and HeLa cells incubated with **59** under normoxic and hypoxic conditions. Adapted with permission from [69]. Copyright (2022) Elsevier.

Probe **59** (Figure 24) represents another notable example of a NIR fluorescent probe designed for hypoxia imaging, featuring a directly attached nitro group as a recognition unit for NTR [69]. Upon selective reduction by NTR, probe **59** exhibited remarkable fluorescence, peaking at 740 nm. This resulted in a 32-fold enhancement in fluorescence intensity and an exceptionally low detection limit of 1.09 ng/mL. Probe **59** was effectively utilized for visualizing hypoxic cancer cells (HeLa, HepG2) and hypoxic tumors in a tumor-bearing mouse model. Furthermore, MTT analysis indicated negligible cytotoxicity, underscoring its potential as a highly valuable tool for both in vitro and in vivo monitoring of hypoxia status.

**Figure 25:**
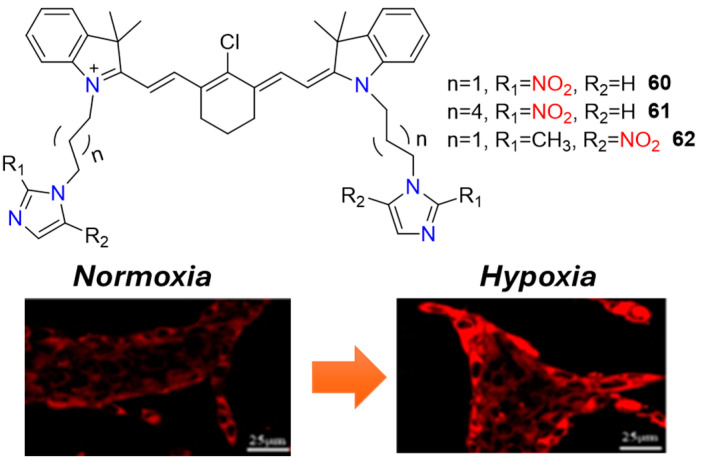
Chemical structures of hypoxia-activated probes **60**–**62** and 4T1 cells incubated with **61** under normoxic and hypoxic conditions. Adapted with permission from [70]. Copyright (2021) American Chemical Society.

The integration of nitroimidazole side chains into NIR fluorophores represents another effective approach for designing turn-on NIR fluorescent hypoxia probes. A notable example includes probes **60**–**62** (Figure 25), where the fluorophore system is linked to two fluorescence-quenching nitroimidazole chains [70]. These probes are designed to preferentially accumulate under hypoxic conditions and exhibit strong fluorescence in the range of 700–900 nm, following the bioreduction of both nitroimidazole fragments. Probes **60**–**62** have been successfully applied for imaging hypoxic tumors both in vivo and in vitro, highlighting their potential utility in hypoxia research and cancer diagnostics.

**Figure 26:**
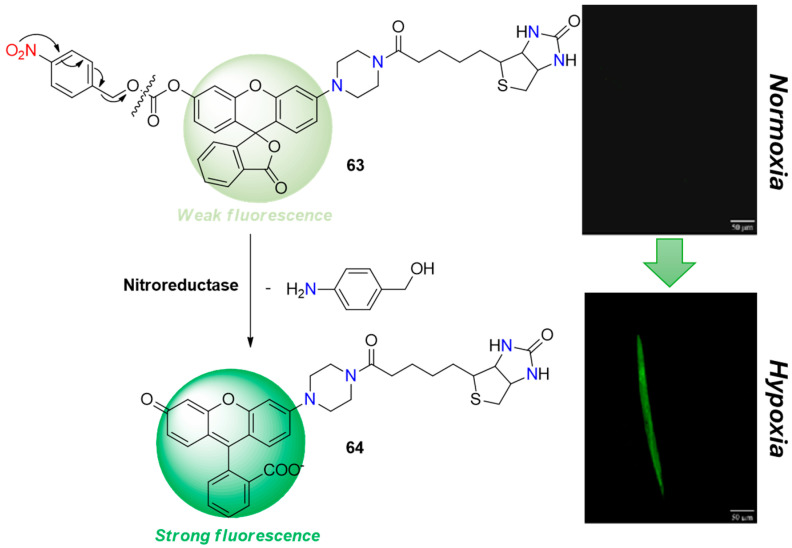
Chemical structures of hypoxia-activated probe **63** and C. elegans incubated with **63** under normoxic and hypoxic conditions. Reproduced with permission from [71]. Copyright (2017) The Royal Society of Chemistry.

The second main approach for designing hypoxic fluorescent probes involves fluorogenic systems that incorporate 4-nitrobenzyl formate, 4-nitrobenzylic, or similar heterocyclic fragments into hypoxic cells, typically binding to amino or hydroxy groups. This approach offers greater flexibility in designing signaling activation compared to the simple reduction of aromatic nitro groups to amines discussed earlier. These probes undergo a scavenging reaction during bioreduction, allowing for a variety of photophysical signaling mechanisms such as ICT, PET (photoinduced electron transfer), and ESIPT (excited-state intramolecular proton transfer). Compound **63** (Figure 26) serves as a classic example of a probe containing 4-nitrobenzyl formate, designed for detecting NTR [71]. It features a rhodolite fluorophore system known for its high quantum yield and stability across a wide pH range. In **63**, the presence of nitrobenzyl formate quenches the rhodol fluorescence, but this quenching is easily reversed in the presence of NTR. Upon reduction, a strong fluorescence emission is observed, facilitating the selective determination of NTR. The fluorescence intensity at 550 nm increases approximately 4.3-fold upon activation, with a reported detection limit of 51.5 ng/mL for NTR. In studies involving Hi-5 cells, compound **63** exhibited non-toxic effects and good cell permeability. It was successfully utilized for imaging hypoxic conditions both in vitro, using Hi-5 cells as a model, and in vivo, in *C. elegans*. 

**Figure 27:**
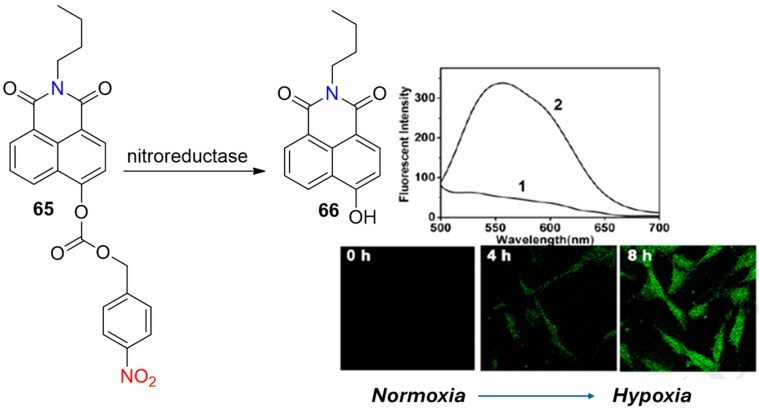
Chemical structures of hypoxia-activated probes **65** and U87 cells incubated with **65** under normoxic (1) and hypoxic (2) conditions. Adapted with permission from [72]. Copyright (2018) Elsevier.

Probe **65,** as reported by Wei et al. (Figure 27), is based on a naphthalimide fluorogenic architecture and serves as another example of the efficient use of the 4-nitrobenzyl formiatic-recognizing unit in the detection of hypoxia [72]. The fluorescence intensity of **65** was increased with the increased concentration of NTR, with a linear range of 0.1–0.3 μg/mL. From the observed standard plot, a detection limit of 0.1 μM was calculated. Furthermore, this probe showed remarkably low cytotoxicity, as even at high concentrations of **65,** the percentage of cell viability remained above 95%. The confocal fluorescence imaging of U87 cells revealed the great potential of the probe to monitor the hypoxic status of tumor cells.

**Figure 28:**
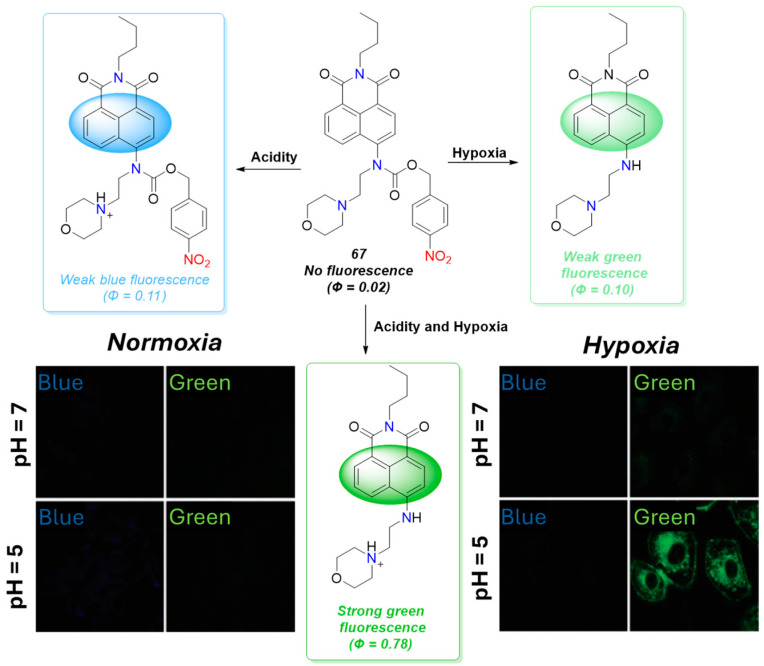
Fluorescence sensing mechanism of probe **67** for detection of acidity and hypoxia, and A549 cells incubated with **67** under normoxic and hypoxic conditions, at different pHs. Adapted with permission from [73]. Copyright (2018) The Royal Society of Chemistry.

The tumor cells were characterized not only by a hypoxic environment but also by increased acidity. That is why the simultaneous detection of acidity and NTR could reduce the possibility of false positive results during intracellular tumor imagining. From this point of view, compound **67** (Figure 28) represents a noteworthy probe for accurate tumor imaging due to its capability to detect both acidity and NTR [73]. In probe **67** a 4-nitrobenzyl formiatic fragment was introduced in a classic PET (photoinduced electron transfer) 4-amino-1,8-naphthalimde pH sensor based on a “fluorophore-spacer-receptor” model where the 4-amino-1,8-naphthalimide serves as the fluorophore, and morpholine acts as the pH receptor. After excitation, electron transfer from the electron-rich morpholine amine to the fluorophore occurs in this molecule, which quenches the fluorescence emission. Upon protonation in acid media, the morpholine formed an electron-poor quaternary ammonium salt, the PET process became impossible, and blue fluorescence was registered. However, the observed fluorescence was weak due to the presence of hypoxia-recognition 4-nitrobenzyl formiatic unit, which quenches emission too. In neutral hypoxic media, the 4-nitrobenzyl formiat in **67** was reduced selectively to form a green-emitting compound. As a result of the reduction-scavenging reaction, the strong electron-accepting carbonyl coupled to the 4-amino nitrogen in 1,8-naphthalimide was cut off. This led to an increase in the electron-donating ability of the 4-amino substituent attached to the 1,8-naphthalimide, thus increasing the fluorophore ICT efficiency and causing the fluorescence emission to red-shift from the blue to the green region. The observed green emission was weak too due to the PET quenching effect in neutral media mentioned above. When the probe is in an acid and hypoxia environment, both quenching processes are blocked, and bright green fluorescence appears. Furthermore, based on both fluorescence outputs (blue at 460 nm and green at 524 nm), a ratiometric analysis was conducted. In ratiometric methods for analyte determination, the quantification is based on the ratio of fluorescent intensities at two different wavelengths. This approach is desirable in bioimaging because it allows for self-calibration and built-in correction for environmental effects and biomolecules. The pH 5 ratiometric analysis for the detection of NTR showed linearity in the range of 0–20 μM and a limit of detection of 0.92 μg/mL. The probe was applied for the fluorescence imaging of acidity and hypoxia in A549 cells.

**Figure 29:**
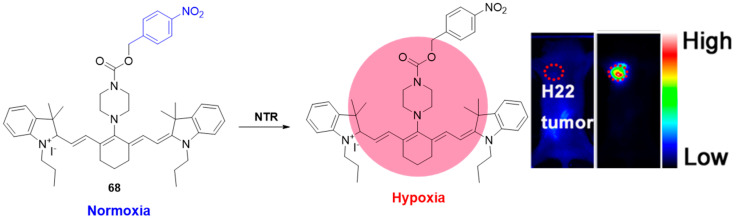
Fluorescence sensing mechanism of probe **68** for detection of hypoxia, and in vivo hypoxia-activated tumor imagining with **68**. Adapted with permission from [74]. Copyright (2018) Elsevier.

Zheng et al. elegantly demonstrated the use of 4-nitrobenzyl formiate as an NTR recognition unit combined with NIR imaging for in vivo hypoxia detection, as shown in their cyanine probe **68** (Figure 29) [74]. The fluorescent analysis of **68** revealed a linear enhancement at 785 nm in the presence of 0–0.5 μg/mL NTR, with a low detection limit (LOD) of 0.0242 μg/mL. The efficient NTR detection capability of **68** was successfully applied for the fluorescence imaging of hypoxic A549, PC-12, and HUVEC cell lines. Furthermore, in vivo hypoxia imaging using probe **68** was evaluated in tumor-bearing mice, as well as in models of cerebral ischemia and deep vein thrombosis. The results underscored the high potential of probe **68** for rapid and precise in vivo monitoring of NTR activity across diverse clinical models.

**Figure 30:**
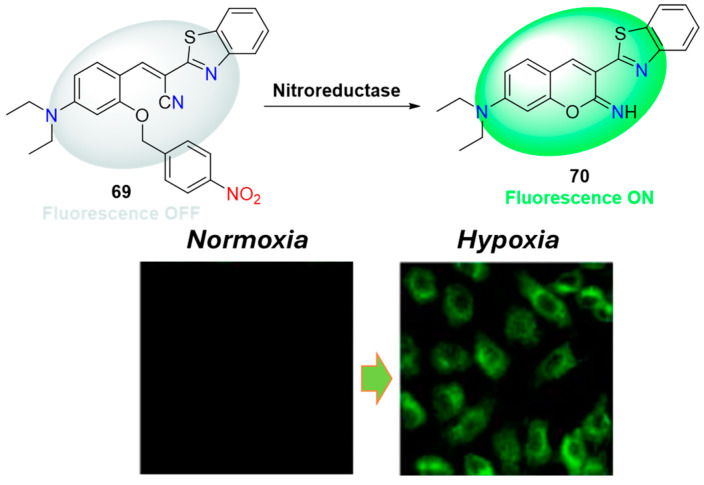
Fluorescence sensing mechanism of probe **69** for detection of NTR and HepG2 cells incubated with **69** under normoxic and hypoxic conditions. Adapted with permission from [75]. Copyright (2018) Elsevier.

Using 4-nitrobenzyl instead of the 4-nitrobenzyl formiate fragment as a recognition unit offers another approach in designing selective probes for detecting NTR and imaging hypoxic cells. Probe **69** (Figure 30) exemplifies this strategy, where the reduction by nitroreductase leads to the formation of a green-emitting fluorescent compound through the scavenging of the 4-nitrobenzylic unit and intramolecular cyclization [75]. Due to the specific interaction of the 4-nitrobenzylic moiety with NTR, compound **69** exhibits increased fluorescence intensity at 530 nm, with a linear response in the concentration range of NTR from 0 to 10 μg/mL and a detection limit of 11 ng/mL. The probe has been successfully used to visualize hypoxic conditions in living HepG2 cells, demonstrating low toxicity and the ability to detect NTR in tumor tissues up to a depth of 100 μm.

**Figure 31:**
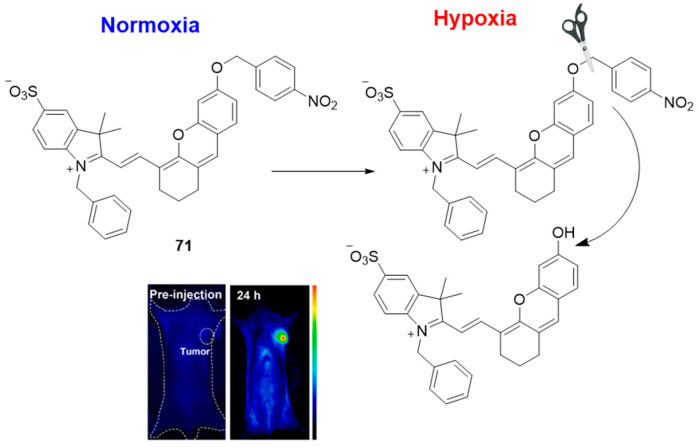
Fluorescence sensing mechanism of probe **71** for detection of hypoxia, and in vivo hypoxia-activated tumor imagining with **71**. Adapted with permission from [76]. Copyright (2022) Elsevier.

**Figure 32:**
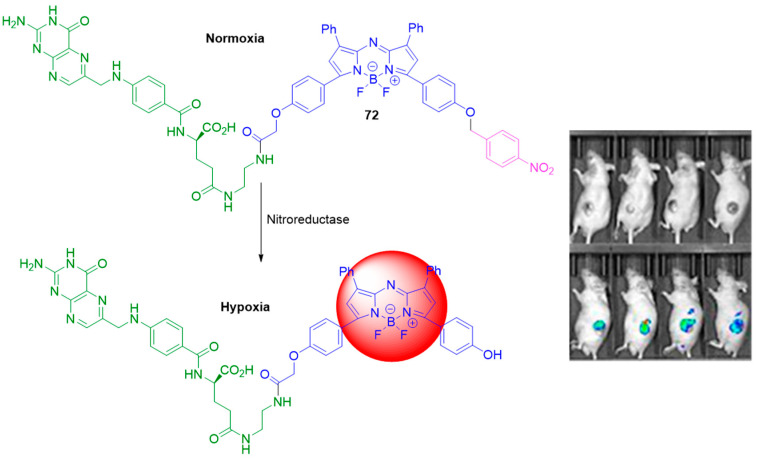
Fluorescence sensing mechanism of probe **72** for detection of hypoxia, and in vivo hypoxia-activated tumor imagining with **72**. Adapted with permission from [77]. Copyright (2021) American Chemical Society.

Figure 31 and Figure 32 illustrate two fluorescent probes (**71** and **72**) as typical examples for in vivo NIR imagining of hypoxic conditions using the NTR-selective 4-nitrobenzyl recognition unit [76,77]. Compound **71** represents a high-efficiency NIR fluorescence probe for the detection of hypoxia via responding to NTR. It showed gradually increased fluorescence at 710 nm in the presence of NTR. The observed LOD was 13.441 ng/mL within the linear range of 0.1–0.9 μg/mL. The in vitro confocal-mediated competitive binding inhibition and flow cytometry indicated a good specificity and sensitivity of **71** toward hypoxic cell detection. The probe showed low toxicity and, more importantly, the in vivo results showed a rapid response in tumor recognition and monitoring of liver cancer, enteritis, and liver ischemia.

Probe **72** is a BODIPY-based fluorogenic compound with quenched emissive properties due to the presence of a 4-nitrobenzyl fragment. The selective reduction of **72** in the presence of NTR results in the formation of a fluorescent compound following the 4-nitrobenzyl scavenging reaction. A 20-fold increase in fluorescent emission was observed after the addition of 1 μg/mL NTR. The detection limit of **72**, calculated according to regression analysis, was found to be 1.52 ng/mL NTR. The probe was nontoxic, with ≥90% cell viability after incubation with 0–50 μM for 24 h. The in vivo NIR optical imaging of CT26 solid tumor-bearing mice suggests that **72** could serve as a tumor-targeting, hypoxia-activatable probe for direct cancer monitoring both in vitro and in vivo.

**Figure 33:**
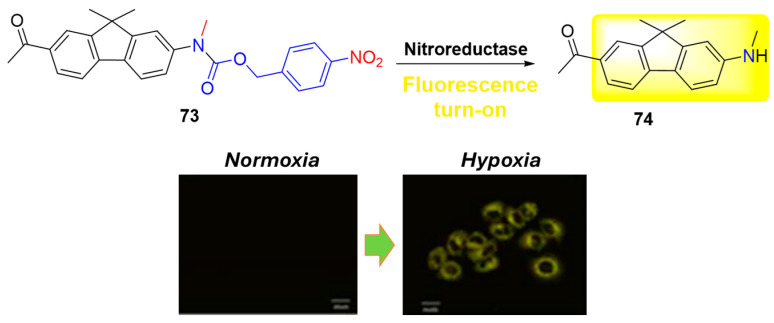
Fluorescence sensing mechanism of probe **73** for detection of NTR and HepG2 cells incubated with **73** under normoxic and hypoxic conditions. Adapted with permission from [78]. Copyright (2017) The Royal Society of Chemistry.

**Figure 34:**
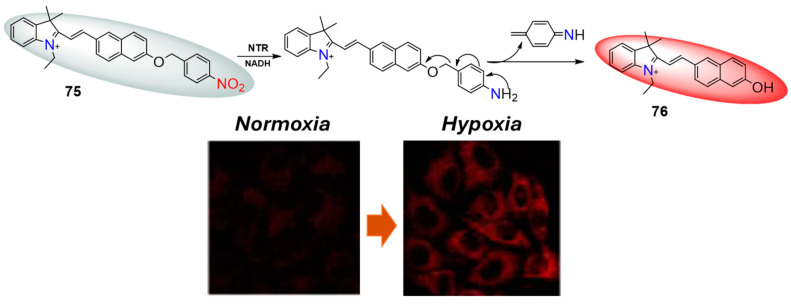
Fluorescence sensing mechanism of probe **75** for detection of NTR and A549 cells incubated with **75** under normoxic and hypoxic conditions. Adapted with permission from [79]. Copyright (2020) Elsevier.

In recent times, there has been significant attention given to two-photon fluorescent probes due to their advantages over traditional one-photon probes, including deeper tissue imaging depth, higher spatial resolution, and longer observation times. This motivated Zhai et al. and Wang et al. to synthesize two-photon probes **73** (Figure 33) and **75** (Figure 34), which incorporate the 4-nitrobenzylic receptor fragment for selective recognition of nitroreductase (NTR) [78,79]. Compound **73** exhibited a remarkable 130-fold fluorescence enhancement at 563 nm within 10 min of reduction by NTR, with a detection limit of 23.67 ng/mL. It was successfully employed for imaging NTR activity in living HeLa cells, tissues, and zebrafish under hypoxic conditions. Notably, in a rat liver tumor model, probe **73** produced bright fluorescence even at a tissue depth of 200 μm, highlighting its effectiveness in deep-tissue imaging scenarios.

Similarly to **73**, probe **75** exhibits significant fluorescence enhancement upon selective reduction by NTR, albeit at a wavelength centered around 580 nm. The quantum yield of fluorescence post-reduction was notably increased to 0.045, compared to only 0.001 in its initial state. There exists a strong linear correlation between the fluorescence enhancement and NTR concentrations within the range of 0–20 μg/mL, with a calculated limit of detection of 26 ng/mL. Probe **75** has been effectively utilized for hypoxia imaging in A549 cell lines and A549 xenograft mice models, demonstrating its practical application in biological settings. Furthermore, MTT assays have revealed low toxicity associated with probe **75**, supporting its potential for safe use in biological and medical research contexts.

**Figure 35:**
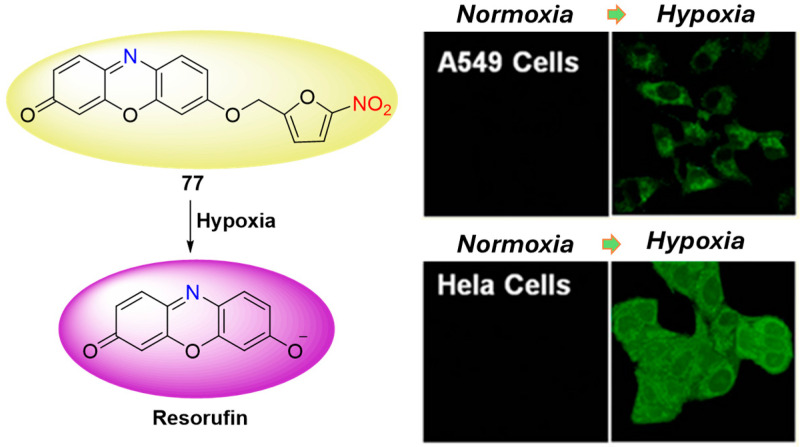
Fluorescence sensing mechanism of probe **77** for detection of NTR and Hela and A549 cells incubated with **77** under normoxic and hypoxic conditions. Adapted with permission from [80]. Copyright (2013) American Chemical Society.

Some nitro heterocycle side chains, similar to the 4-nitrobenzylic and 4-nitrobenzyl formiatic units discussed earlier, interact with NTR through scavenger reduction, making them promising recognition components in the design of fluorescent probes for detecting and imaging NTR. For example, phenoxazinone **77,** depicted in Figure 35, contains a 5-nitrofuranyl moiety that undergoes scavenger reduction, resulting in the formation of a highly fluorescent compound after the furane group is removed [80]. This reduction process is selective to NTR and enables its fluorescent detection. Probe **77** exhibits a remarkable 100-fold fluorescence enhancement and demonstrates a limit of detection of 0.27 ng/mL. Its potential for tumor diagnosis via hypoxia imaging was demonstrated by monitoring the hypoxic status in HeLa and A549 cells. Furthermore, standard MTT assays revealed that cell viability remained unaffected even at high concentrations (up to 5 μM) of **77**, indicating its low toxicity profile.

**Figure 36:**
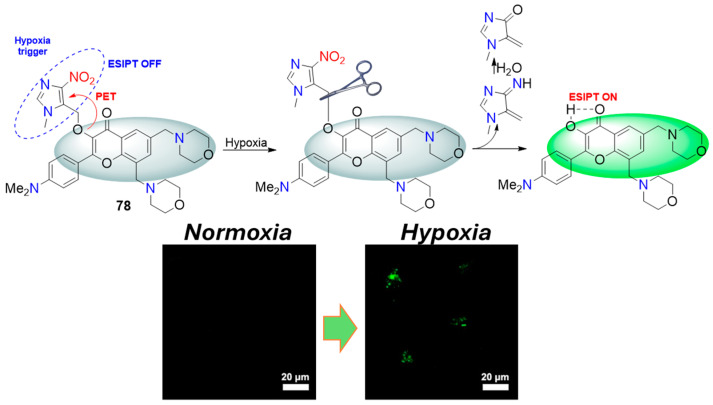
Fluorescence sensing mechanism of probe **78** for detection of NTR and Hela cells incubated with **78** under normoxic and hypoxic conditions. Adapted with permission from [81]. Copyright (2016) Elsevier.

Probe **78**, as reported by Feng et al. (Figure 36), is another interesting example in which a 4-nitroimidazole recognition unit could be selectively removed after reduction with nitroreductase [81]. Probe **78** itself is nonfluorescent due to the photoinduced electron transfer to the nitroimidazole. However, after reduction by NTR, it was converted to 4-hydroxy-3-hydroxyflavone, which exhibits bright fluorescence at 560 nm due to ESIPT (intramolecular proton transfer). The ESIPT process showed an unusually high Stokes shift, which is a serious advantage in fluorescence sensing measurements, especially in living systems, because it could reduce the influence of unwanted self-reabsorption and the inner-filter effect. Due to the higher sensitivity and selective turn-on fluorescence response, probe **78** showed high potential for the detection of NTR, with good linearity in the concentration range of NTR 1–4 μg/mL and a limit of detection of 63 ng/mL. In addition, this probe displayed low cytotoxicity, good biocompatibility, and was successfully applied for imaging the hypoxic status of HeLa cells.

**Figure 37:**
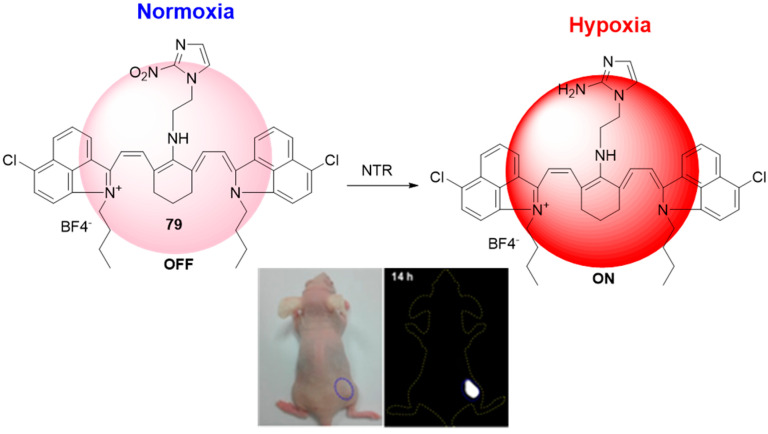
Fluorescence sensing mechanism of probe **79** for detection of hypoxia, and in vivo hypoxia-activated tumor imagining with **79**. Adapted with permission from [82]. Copyright (2018) Ivyspring International Publisher.

In the last decade, the design and synthesis of selectively activated fluorescent probes in the second near-infrared window (NIR-II) has focused in general on the in vivo imagining of various biological or pathological processes, since NIR-II fluorescence imaging has an improved penetration depth and reduced autofluorescence. This motivated Meng et. al. to fabricate molecular probe **79** (Figure 37), which exhibits weak fluorescence at the NIR-II region due to the presence of a nitroimidazole fluorescent quencher [82]. The selective reduction of the nitro group to an amine in the presence of NTR resulted in a 107-fold fluorescent enhancement at 1046 nm. The NIR-II fluorescence signal of **78** in the tumor tissue was clearly visible at 10 h post-injection and reached its maximum at about 14 h. Notably, the in vivo NIR-II fluorescence imaging of tumor hypoxia with 78 showed an unusual lack of observable background signal. The probe itself was safe, and it was found that it could be eliminated by renal excretion pathways from the animal body. All observed results revealed the great importance of probe **79** as a promising contrast and theranostic agent for hypoxia-related diseases, such as cancer, inflammation, stroke, and cardiac ischemia.

**Figure 38:**
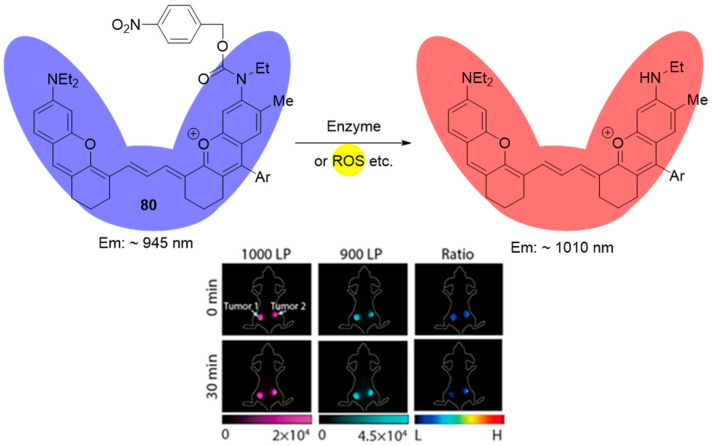
Fluorescence sensing mechanism of probe **80** for detection of hypoxia, and in vivo hypoxia-activated tumor imagining with **80**. Adapted with permission from [83]. Copyright (2022) American Chemical Society.

Karan et. al. reported another NIR II fluorescent probe (**80**) using 4-nitrobenzyl fragment as the NTR recognition element [83]. This probe was characterized by selective and a 4-fold more intensive ratiometric (1000 nm/940 nm) fluorescent output after activation by scavenging reduction of the 4-nitrobenzyl moiety. The ratiometric fluorescent response takes 45 min to reach its maximal value. It shows an excellent linear relationship against the concentrations of NTR from 0 to 10 μg/mL. The in vivo study indicated that probe **80** could be used for the visualization of tumor tissues due to the selective activation by NTR. Female BALB/c mice bearing two subcutaneous 4T1 breast tumors were chosen as a model. This probe offers a valuable in vivo biosensing amalgamation of NIR-II fluorescent response and self-calibrated ratiometric analysis (see Figure 38).

## 5. Conclusions

The heterogeneity of tumor hypoxia, pharmacokinetics, and the potential toxicity of conventional drugs remain significant challenges for HAPs. Continued research into nitro(het)aromatic compounds and their structural modifications is essential for developing more selective and effective cancer therapies. This review underscores the potential of hypoxia-activated nitro(het)aromatic prodrugs in targeted cancer therapy, particularly through mechanisms such as gene-directed enzyme prodrug therapy and the formulation of bioreductive-activated prodrug conjugates featuring nitrophenyl, nitrobenzyl, and nitroheteroaryl triggers. While promising, some BAPCs require further refinement to reduce activation in normoxic conditions and improve water solubility. To address these challenges, the design of next-generation hypoxia-activated pro-drugs focuses on incorporating bulkier nitroaromatic groups and triggers with higher reduction potentials, which better match the cellular reductase range. This approach aims to improve specificity and minimize the off-target effects, thereby maximizing drug delivery and activation specifically within hypoxic tumor regions. Overall, BAPCs represent a significant advancement in targeted cancer therapy, offering a method to specifically deliver and activate drugs in hypoxic tumor regions, thereby enhancing efficacy and minimizing systemic toxicity. Stability and bioavailability issues arise from poor compound stability in biological environments and limited bioavailability. To address these, chemical modifications to enhance stability and formulation strategies, including encapsulation in liposomes or other nanocarriers, are recommended.

Over the past four decades, nitroaromatic compounds have played a crucial role in the design and synthesis of fluorescent probes for the selective detection of NTR and for imaging the hypoxic status of tumor cells and tissues. The major appeal of nitroaromatic derivatives lies in their simple synthesis and predictable off-on fluorescent response after selective bioreduction by NTR. Many of the developed probes exhibit low toxicity, high sensitivity, and linearity in detection ranges, making them suitable for in vivo applications in the hypoxia imaging of tumors. Despite significant progress, enhancing water solubility remains a critical hurdle due to the inherently hydrophobic nature of most organic architectures, limiting their practical application.

Future research directions include developing multianalyte probes capable of detecting acidity and NTR simultaneously, thereby reducing false positives in clinical settings. Moreover, exploring two-photon fluorescent technologies for deeper tissue imaging and improved diagnostic accuracy holds promise. These advancements highlight the potential of nitro(het)aromatic compounds in revolutionizing cancer diagnostics and therapy, offering reliable, non-invasive solutions for early cancer detection and treatment monitoring.

We hope this review will assist researchers in creating novel nitro(het)aromatic compounds for treating with improve selectivity and stability, and/or for fluorescently imaging hypoxic tumors to bolster cancer diagnostics and improve the monitoring of treatment efficacy.

## Data Availability

Data are available within the article.
